# *N*-Benzyl-*N*-ethyl-4-methyl­benzene­sulfonamide

**DOI:** 10.1107/S1600536810035105

**Published:** 2010-09-04

**Authors:** Islam Ullah Khan, Waqar Ahmad, Muhammad Nadeem Arshad, Shahzad Sharif, Jamil Ahmed

**Affiliations:** a Materials Chemistry Laboratory, Department of Chemistry, GC University, Lahore 54000, Pakistan

## Abstract

In the title compound, C_16_H_19_NO_2_S, the dihedral angle between the two aromatic rings is 84.78 (7)°. Weak inter­molecular C—H⋯O inter­actions stabilize the crystal structure by the formation of a 16-membered *R*_2_^2^(16) ring motif.

## Related literature

For biological activity of sulfonamides, see: Maren (1976 ▶); Boyd (1988 ▶). For a related structure, see: Khan *et al.* (2010 ▶). For graph-set notation, see: Bernstein *et al.* (1995 ▶).
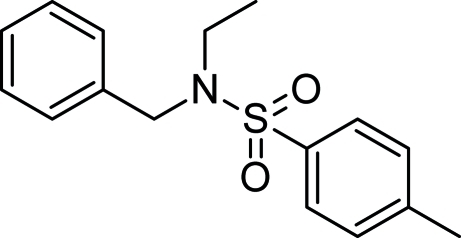


## Experimental

### 

#### Crystal data

C_16_H_19_NO_2_S
*M* *_r_* = 289.38
Monoclinic, 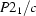

*a* = 8.8144 (3) Å
*b* = 19.7677 (6) Å
*c* = 9.8914 (4) Å
β = 117.689 (1)°
*V* = 1526.11 (9) Å^3^
*Z* = 4
Mo *K*α radiation
μ = 0.21 mm^−1^
*T* = 296 K
0.41 × 0.25 × 0.19 mm

#### Data collection

Bruker Kappa APEXII CCD diffractometer
Absorption correction: multi-scan (*SADABS*; Bruker, 2007 ▶) *T*_min_ = 0.918, *T*_max_ = 0.961
13763 measured reflections
3489 independent reflections
2168 reflections with *I* > 2σ(*I*)
*R*_int_ = 0.036

#### Refinement

*R*[*F* ^2^ > 2σ(*F* ^2^)] = 0.046
*wR*(*F* ^2^) = 0.125
*S* = 1.02
3489 reflections
183 parameters
H-atom parameters constrained
Δρ_max_ = 0.21 e Å^−3^
Δρ_min_ = −0.21 e Å^−3^

### 

Data collection: *APEX2* (Bruker, 2007 ▶); cell refinement: *SAINT* (Bruker, 2007 ▶); data reduction: *SAINT*; program(s) used to solve structure: *SHELXS97* (Sheldrick, 2008 ▶); program(s) used to refine structure: *SHELXL97* (Sheldrick, 2008 ▶); molecular graphics: *ORTEP-3 for Windows* (Farrugia, 1997 ▶) and *PLATON* (Spek, 2009 ▶); software used to prepare material for publication: *WinGX* (Farrugia, 1999 ▶) and *PLATON*.

## Supplementary Material

Crystal structure: contains datablocks I, global. DOI: 10.1107/S1600536810035105/bt5337sup1.cif

Structure factors: contains datablocks I. DOI: 10.1107/S1600536810035105/bt5337Isup2.hkl

Additional supplementary materials:  crystallographic information; 3D view; checkCIF report

## Figures and Tables

**Table 1:** Hydrogen-bond geometry (Å, °)

*D*—H⋯*A*	*D*—H	H⋯*A*	*D*⋯*A*	*D*—H⋯*A*
C7—H7*C*⋯O1^i^	0.96	2.58	3.354 (3)	138

Symmetry code: (i) 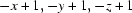.

**Fig. 1.**
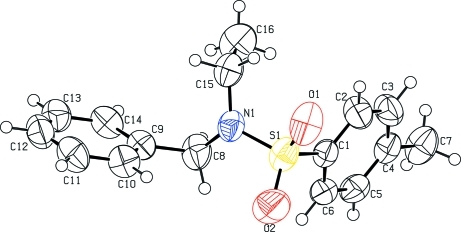
The structure of the title compound with labeling and displacement ellipsoids drawn at the 50% probability level.

**Fig. 2.**
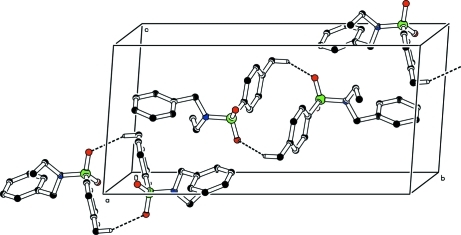
Unit cell packing with hydrogen bonding shown as dashed lines.

## supplementary crystallographic information

### Comment

Sulfonamides have extensively been reported for their wide variety of pharmacological activities such as antibacterial (Maren, 1976) and diuretic (Boyd, 1988). The present structure is countinous to our previous reported derivative of sulfonamide (Khan *et al.*, 2010).

The two aromatic rings   in the title sulfonamide molecule are inclined to each other at an angle of 84.78(0.07)°. The C–H···O type weak intermolecular hydrogen bonding between the C–H of methyl group and oxygen of the SO_2_ forms the dimers which result in the formation of 16 membered ring motif which can be represented as *R*_2_^2^(16) in graph set patterns (Bernstein *et al.*, 1995).

### Experimental

A mixture of N-benzyl-4-methylbenzenesulfonamide (0.5 g, 2.02mmol) and sodium hydride (0.2 g, 8.333 mmol) in N, N dimethylformamide (10 ml) was stirred at room temperature for 30 minutes followed by the addition of ethyl iodide (0.62 ml 2.02mmol) After the consumption of reactants (as monitored by TLC), the contents were poured over crushed ice. The precipitated product was isolated, washed, dried and recrystallized from methanol solution to yield colorless blocks of title compound.

### Refinement

All the C-H H-atoms were positioned geometricaly with C—H = 0.93 Å for aromatic, C—H = 0.96 Å for CH_3_, C—H = 0.97 Å for CH_2_ and were refined using a riding model with *U*_iso_(H) = 1.2 *U*_eq_ for aromatic and CH_2_ and with *U*_iso_(H) = 1.5 *U*_eq_ for CH_3_ Carbon atoms. The two reflection (0 2 0 and 1 1 0) were omitted in final refinement as these were obscured by the beam stop.

### Crystal data

**Table:**

C_16_H_19_NO_2_S	*F*(000) = 616
*M**_r_* = 289.38	*D*_x_ = 1.259 Mg m^−^^3^
Monoclinic, *P*2_1_/*c*	Mo *K*α radiation, λ = 0.71073 Å
Hall symbol: -P 2ybc	Cell parameters from 3170 reflections
*a* = 8.8144 (3) Å	θ = 2.5–22.0°
*b* = 19.7677 (6) Å	µ = 0.21 mm^−^^1^
*c* = 9.8914 (4) Å	*T* = 296 K
β = 117.689 (1)°	Needle, colorless
*V* = 1526.11 (9) Å^3^	0.41 × 0.25 × 0.19 mm
*Z* = 4	

### Data collection

**Table:**

Bruker Kappa APEXII CCD diffractometer	3489 independent reflections
Radiation source: fine-focus sealed tube	2168 reflections with *I* > 2σ(*I*)
graphite	*R*_int_ = 0.036
φ and ω scans	θ_max_ = 27.5°, θ_min_ = 3.3°
Absorption correction: multi-scan (*SADABS*; Bruker, 2007)	*h* = −11→11
*T*_min_ = 0.918, *T*_max_ = 0.961	*k* = −25→24
13763 measured reflections	*l* = −11→12

### Refinement

**Table:**

Refinement on *F*^2^	Primary atom site location: structure-invariant direct methods
Least-squares matrix: full	Secondary atom site location: difference Fourier map
*R*[*F*^2^ > 2σ(*F*^2^)] = 0.046	Hydrogen site location: inferred from neighbouring sites
*wR*(*F*^2^) = 0.125	H-atom parameters constrained
*S* = 1.02	*w* = 1/[σ^2^(*F*_o_^2^) + (0.0529*P*)^2^ + 0.2532*P*] where *P* = (*F*_o_^2^ + 2*F*_c_^2^)/3
3489 reflections	(Δ/σ)_max_ < 0.001
183 parameters	Δρ_max_ = 0.21 e Å^−^^3^
0 restraints	Δρ_min_ = −0.20 e Å^−^^3^

### Special details

**Table:**

Geometry. All s.u.'s (except the s.u. in the dihedral angle between two l.s. planes) are estimated using the full covariance matrix. The cell s.u.'s are taken into account individually in the estimation of s.u.'s in distances, angles and torsion angles; correlations between s.u.'s in cell parameters are only used when they are defined by crystal symmetry. An approximate (isotropic) treatment of cell s.u.'s is used for estimating s.u.'s involving l.s. planes.
Refinement. Refinement of F^2^ against ALL reflections. The weighted R-factor wR and goodness of fit S are based on F^2^, conventional R-factors R are based on F, with F set to zero for negative F^2^. The threshold expression of F^2^ > 2σ(F^2^) is used only for calculating R-factors(gt) etc. and is not relevant to the choice of reflections for refinement. R-factors based on F^2^ are statistically about twice as large as those based on F, and R- factors based on ALL data will be even larger.

### Fractional atomic coordinates and isotropic or equivalent isotropic displacement parameters (Å^2^)

**Table:**

	*x*	*y*	*z*	*U*_iso_*/*U*_eq_	
S1	0.70800 (8)	0.37535 (3)	0.46668 (7)	0.0682 (2)	
O1	0.6074 (2)	0.39018 (8)	0.30736 (18)	0.0865 (5)	
O2	0.8768 (2)	0.40136 (9)	0.5483 (2)	0.1020 (6)	
N1	0.7241 (2)	0.29352 (8)	0.48024 (18)	0.0612 (4)	
C1	0.5929 (2)	0.40249 (9)	0.5623 (2)	0.0517 (5)	
C2	0.4177 (3)	0.40689 (11)	0.4843 (2)	0.0692 (6)	
H2	0.3605	0.3980	0.3801	0.083*	
C3	0.3266 (3)	0.42449 (12)	0.5602 (3)	0.0716 (6)	
H3	0.2078	0.4270	0.5063	0.086*	
C4	0.4073 (3)	0.43849 (9)	0.7140 (2)	0.0581 (5)	
C5	0.5832 (3)	0.43392 (10)	0.7901 (2)	0.0618 (5)	
H5	0.6405	0.4431	0.8942	0.074*	
C6	0.6766 (3)	0.41622 (10)	0.7163 (2)	0.0599 (5)	
H6	0.7954	0.4135	0.7701	0.072*	
C7	0.3065 (3)	0.45867 (12)	0.7953 (3)	0.0836 (7)	
H7A	0.3692	0.4465	0.9011	0.125*	
H7B	0.1981	0.4358	0.7501	0.125*	
H7C	0.2880	0.5067	0.7867	0.125*	
C8	0.8382 (3)	0.26352 (12)	0.6291 (2)	0.0743 (6)	
H8A	0.7710	0.2490	0.6785	0.089*	
H8B	0.9191	0.2974	0.6934	0.089*	
C9	0.9347 (2)	0.20401 (10)	0.6128 (2)	0.0564 (5)	
C10	1.0400 (3)	0.21151 (12)	0.5454 (2)	0.0667 (6)	
H10	1.0521	0.2537	0.5099	0.080*	
C11	1.1276 (3)	0.15629 (14)	0.5306 (3)	0.0765 (7)	
H11	1.1990	0.1617	0.4856	0.092*	
C12	1.1105 (3)	0.09461 (13)	0.5810 (3)	0.0761 (7)	
H12	1.1692	0.0578	0.5697	0.091*	
C13	1.0084 (3)	0.08631 (12)	0.6478 (3)	0.0735 (6)	
H13	0.9975	0.0439	0.6830	0.088*	
C14	0.9203 (3)	0.14070 (12)	0.6639 (2)	0.0667 (6)	
H14	0.8502	0.1346	0.7098	0.080*	
C15	0.5806 (3)	0.25212 (11)	0.3735 (2)	0.0660 (6)	
H15A	0.6267	0.2131	0.3454	0.079*	
H15B	0.5157	0.2783	0.2814	0.079*	
C16	0.4591 (3)	0.22731 (13)	0.4301 (3)	0.0914 (8)	
H16A	0.3641	0.2047	0.3487	0.137*	
H16B	0.4178	0.2650	0.4648	0.137*	
H16C	0.5177	0.1963	0.5130	0.137*	

### Atomic displacement parameters (Å^2^)

**Table:**

	*U*^11^	*U*^22^	*U*^33^	*U*^12^	*U*^13^	*U*^23^
S1	0.0794 (4)	0.0589 (3)	0.0904 (4)	−0.0003 (3)	0.0599 (3)	−0.0035 (3)
O1	0.1339 (15)	0.0741 (10)	0.0837 (11)	0.0164 (9)	0.0777 (11)	0.0182 (8)
O2	0.0814 (11)	0.0933 (13)	0.1601 (17)	−0.0224 (10)	0.0802 (12)	−0.0314 (12)
N1	0.0701 (11)	0.0557 (10)	0.0617 (10)	0.0093 (8)	0.0339 (9)	−0.0037 (8)
C1	0.0571 (11)	0.0439 (10)	0.0604 (12)	0.0018 (8)	0.0325 (10)	−0.0018 (9)
C2	0.0655 (14)	0.0854 (16)	0.0522 (11)	0.0167 (11)	0.0236 (11)	−0.0053 (10)
C3	0.0549 (12)	0.0855 (16)	0.0723 (14)	0.0125 (11)	0.0278 (11)	−0.0083 (12)
C4	0.0703 (13)	0.0458 (11)	0.0710 (13)	0.0002 (9)	0.0436 (11)	−0.0032 (9)
C5	0.0741 (14)	0.0562 (12)	0.0551 (11)	−0.0053 (10)	0.0300 (11)	−0.0099 (9)
C6	0.0533 (11)	0.0537 (12)	0.0702 (13)	−0.0035 (9)	0.0266 (10)	−0.0108 (10)
C7	0.1066 (19)	0.0724 (15)	0.1055 (18)	−0.0004 (13)	0.0777 (16)	−0.0079 (13)
C8	0.0868 (16)	0.0822 (16)	0.0593 (13)	0.0194 (13)	0.0386 (12)	−0.0065 (11)
C9	0.0559 (11)	0.0698 (13)	0.0473 (10)	0.0052 (10)	0.0272 (9)	−0.0012 (9)
C10	0.0695 (13)	0.0730 (14)	0.0675 (13)	0.0003 (11)	0.0402 (12)	0.0072 (11)
C11	0.0620 (13)	0.1046 (19)	0.0791 (15)	0.0116 (13)	0.0465 (12)	0.0010 (14)
C12	0.0651 (14)	0.0816 (17)	0.0751 (15)	0.0202 (12)	0.0269 (12)	−0.0006 (13)
C13	0.0690 (14)	0.0671 (15)	0.0748 (15)	0.0079 (11)	0.0253 (12)	0.0121 (11)
C14	0.0596 (12)	0.0867 (16)	0.0601 (12)	0.0024 (11)	0.0332 (11)	0.0094 (11)
C15	0.0831 (15)	0.0625 (13)	0.0650 (13)	0.0064 (11)	0.0450 (12)	0.0010 (10)
C16	0.107 (2)	0.0864 (18)	0.108 (2)	−0.0098 (15)	0.0732 (18)	−0.0064 (14)

### Geometric parameters (Å, °)

**Table:**

S1—O2	1.4192 (18)	C8—C9	1.504 (3)
S1—O1	1.4342 (17)	C8—H8A	0.9700
S1—N1	1.6239 (17)	C8—H8B	0.9700
S1—C1	1.7614 (18)	C9—C14	1.378 (3)
N1—C15	1.464 (3)	C9—C10	1.379 (3)
N1—C8	1.468 (3)	C10—C11	1.384 (3)
C1—C2	1.372 (3)	C10—H10	0.9300
C1—C6	1.376 (3)	C11—C12	1.352 (3)
C2—C3	1.374 (3)	C11—H11	0.9300
C2—H2	0.9300	C12—C13	1.352 (3)
C3—C4	1.375 (3)	C12—H12	0.9300
C3—H3	0.9300	C13—C14	1.378 (3)
C4—C5	1.376 (3)	C13—H13	0.9300
C4—C7	1.503 (3)	C14—H14	0.9300
C5—C6	1.375 (3)	C15—C16	1.503 (3)
C5—H5	0.9300	C15—H15A	0.9700
C6—H6	0.9300	C15—H15B	0.9700
C7—H7A	0.9600	C16—H16A	0.9600
C7—H7B	0.9600	C16—H16B	0.9600
C7—H7C	0.9600	C16—H16C	0.9600
			
O2—S1—O1	120.04 (11)	C9—C8—H8A	109.3
O2—S1—N1	106.58 (10)	N1—C8—H8B	109.3
O1—S1—N1	106.27 (9)	C9—C8—H8B	109.3
O2—S1—C1	107.28 (10)	H8A—C8—H8B	108.0
O1—S1—C1	108.16 (10)	C14—C9—C10	118.19 (19)
N1—S1—C1	108.01 (9)	C14—C9—C8	121.22 (18)
C15—N1—C8	117.21 (18)	C10—C9—C8	120.6 (2)
C15—N1—S1	118.93 (14)	C9—C10—C11	120.0 (2)
C8—N1—S1	118.57 (14)	C9—C10—H10	120.0
C2—C1—C6	119.62 (18)	C11—C10—H10	120.0
C2—C1—S1	119.75 (15)	C12—C11—C10	120.6 (2)
C6—C1—S1	120.56 (15)	C12—C11—H11	119.7
C1—C2—C3	119.99 (19)	C10—C11—H11	119.7
C1—C2—H2	120.0	C11—C12—C13	120.2 (2)
C3—C2—H2	120.0	C11—C12—H12	119.9
C2—C3—C4	121.5 (2)	C13—C12—H12	119.9
C2—C3—H3	119.3	C12—C13—C14	120.0 (2)
C4—C3—H3	119.3	C12—C13—H13	120.0
C3—C4—C5	117.64 (18)	C14—C13—H13	120.0
C3—C4—C7	121.0 (2)	C9—C14—C13	120.92 (19)
C5—C4—C7	121.4 (2)	C9—C14—H14	119.5
C6—C5—C4	121.76 (19)	C13—C14—H14	119.5
C6—C5—H5	119.1	N1—C15—C16	116.12 (17)
C4—C5—H5	119.1	N1—C15—H15A	108.3
C5—C6—C1	119.52 (19)	C16—C15—H15A	108.3
C5—C6—H6	120.2	N1—C15—H15B	108.3
C1—C6—H6	120.2	C16—C15—H15B	108.3
C4—C7—H7A	109.5	H15A—C15—H15B	107.4
C4—C7—H7B	109.5	C15—C16—H16A	109.5
H7A—C7—H7B	109.5	C15—C16—H16B	109.5
C4—C7—H7C	109.5	H16A—C16—H16B	109.5
H7A—C7—H7C	109.5	C15—C16—H16C	109.5
H7B—C7—H7C	109.5	H16A—C16—H16C	109.5
N1—C8—C9	111.52 (15)	H16B—C16—H16C	109.5
N1—C8—H8A	109.3		
			
O2—S1—N1—C15	−162.75 (15)	C7—C4—C5—C6	−179.28 (19)
O1—S1—N1—C15	−33.63 (16)	C4—C5—C6—C1	−0.1 (3)
C1—S1—N1—C15	82.24 (15)	C2—C1—C6—C5	0.3 (3)
O2—S1—N1—C8	43.62 (18)	S1—C1—C6—C5	−176.62 (15)
O1—S1—N1—C8	172.75 (15)	C15—N1—C8—C9	65.8 (2)
C1—S1—N1—C8	−71.39 (17)	S1—N1—C8—C9	−140.15 (16)
O2—S1—C1—C2	156.23 (18)	N1—C8—C9—C14	−120.8 (2)
O1—S1—C1—C2	25.4 (2)	N1—C8—C9—C10	58.9 (3)
N1—S1—C1—C2	−89.22 (18)	C14—C9—C10—C11	0.0 (3)
O2—S1—C1—C6	−26.89 (19)	C8—C9—C10—C11	−179.8 (2)
O1—S1—C1—C6	−157.72 (16)	C9—C10—C11—C12	0.4 (3)
N1—S1—C1—C6	87.66 (17)	C10—C11—C12—C13	−0.6 (4)
C6—C1—C2—C3	−0.4 (3)	C11—C12—C13—C14	0.4 (3)
S1—C1—C2—C3	176.47 (17)	C10—C9—C14—C13	−0.1 (3)
C1—C2—C3—C4	0.5 (4)	C8—C9—C14—C13	179.61 (19)
C2—C3—C4—C5	−0.3 (3)	C12—C13—C14—C9	−0.1 (3)
C2—C3—C4—C7	179.1 (2)	C8—N1—C15—C16	57.2 (2)
C3—C4—C5—C6	0.2 (3)	S1—N1—C15—C16	−96.8 (2)

### Hydrogen-bond geometry (Å, °)

**Table:**

*D*—H···*A*	*D*—H	H···*A*	*D*···*A*	*D*—H···*A*
C7—H7C···O1^i^	0.96	2.58	3.354 (3)	138

Symmetry codes: (i) −*x*+1, −*y*+1, −*z*+1.
